# The interaction between individualism and wellbeing in predicting mortality: Survey of Health Ageing and Retirement in Europe

**DOI:** 10.1007/s10865-017-9871-x

**Published:** 2017-07-15

**Authors:** Judith A. Okely, Alexander Weiss, Catharine R. Gale

**Affiliations:** 1 Centre for Cognitive Ageing and Cognitive Epidemiology, Department of Psychology, University of Edinburgh, Edinburgh, UK; 2 Department of Psychology, University of Edinburgh, Edinburgh, UK; 3 MRC Lifecourse Epidemiology Unit, University of Southampton, Southampton, UK

**Keywords:** Wellbeing, Individualism, Longevity, Self-rated health, Cultural dimensions

## Abstract

The link between greater wellbeing and longevity is well documented. The aim of the current study was to test whether this association is consistent across individualistic and collectivistic cultures. The sample consisted of 13,596 participants from 11 European countries, each of which was assigned an individualism score according to Hofstede et al.'s (Cultures and organizations: software of the mind, McGraw Hill, New York, 2010) cultural dimension of individualism. We tested whether individualism moderated the cross-sectional association between wellbeing and self-rated health or the longitudinal association between wellbeing and mortality risk. Our analysis revealed a significant interaction between individualism and wellbeing such that the association between wellbeing and self-rated health or risk of mortality from cardiovascular disease was stronger in more individualistic countries. However, the interaction between wellbeing and individualism was not significant in analysis predicting all-cause mortality. Further prospective studies are needed to confirm our finding and to explore the factors responsible for this culturally dependent effect.

**Electronic supplementary material:**

The online version of this article (doi:10.1007/s10865-017-9871-x) contains supplementary material, which is available to authorized users.

## Introduction

Numerous studies document links between wellbeing or positive affect and favorable health outcomes (Boehm et al., 2011; Chida & Steptoe, 2008; Feller et al., 2013; Martín-María et al., 2017; Wakai et al., 2007). What is, however, unclear, is the extent to which these associations are largely consistent across cultures versus varying across cultures that differ in cultural dimensions related to social networks.

Culture can be defined as a shared set of values, beliefs or behaviours that differentiate one society from another (Hofstede et al., 1991). Matusitz and Musambira (2013) note that “[Culture] is deeply embedded in the unconscious minds, which implies that shared values are not always rational” (p. 45). One dimension used to describe cultural differences is that of individualism/collectivism. In relatively collectivistic cultures, such as China, social interdependence and group loyalty is valued highly. On the other hand, in relatively individualistic cultures, such as the United States, people prioritise their personal interests over those of the wider group into which they are born (Hofstede, 2010).

The degree to which cultures are individualistic versus collectivistic may moderate the association between wellbeing and health. Comparisons of cultures has revealed that individualism/collectivism is associated with the way in which wellbeing is appraised by individuals. For instance, people in individualistic cultures, prioritise positive emotions and personal wellbeing (Ahuvia, 2002; Diener & Suh, 2000; Steptoe et al., 2007; Veenhoven, 1999) and view negative emotions as harmful and undesirable (Wierzbicka, 1994). By contrast, people in more collectivistic cultures acknowledge the importance of experiencing both positive and negative emotions, and value emotional stability rather than positive affect (Lu, 2001; Ng et al., 2003). Comparisons between more individualistic and more collectivistic cultures has also revealed that, in more individualistic cultures, the wellbeing of individuals is most strongly related to their self-esteem and sense of personal achievement, and that, in more collectivistic cultures, wellbeing is most strongly related to interpersonal goals and being able avoid social conflict (Uchida & Oishi, 2016). The relationship between positive and negative affect is also culturally dependent. Specifically, the size of the inverse association between positive affect and depressive symptoms is stronger in individualistic than in collectivistic cultures (Leu et al., 2011).

The idea that culturally-dependent appraisals of emotion might moderate the association between emotions and health has been discussed in the context of negative affect. Two studies tested whether the strength of association between negative affect and health (the number of chronic conditions or levels of inflammatory biomarkers) differed in American and Japanese samples (Curhan et al., 2014; Miyamoto et al., 2013). Both Curhan et al. (2014) and Miyamoto et al. (2013) found that the association between negative affect and health was stronger among American participants. Based on their findings, these two groups of authors concluded that the American tendency to conceptualize negative affect as harmful and a personal responsibility, may cause individuals that experience frequent negative affect to experience additional distress, which, consequently, leads to poorer physical health (Collins et al., 2009; Rugulies, 2002; Saz & Dewey, 2001).

Cultural differences in the evaluation of wellbeing could also impact the link between wellbeing and health. Specifically, a more positive evaluation of high wellbeing in individualistic cultures (Ahuvia, 2002; Diener & Suh, 2000; Steptoe et al., 2007; Veenhoven, 1999) may confer greater health benefits in these cultures via the association between positive affect and both improved physiological functioning (Steptoe & Wardle, 2005) and healthier lifestyle choices (Grant et al. 2009). Furthermore, an emphasis on personal wellbeing in individualistic cultures may cause individuals with low wellbeing to feel distressed (Leu et al., 2011), which may impact negatively these individuals’ health. A more negative appraisal of low wellbeing in individualist cultures may also result in harmful coping practices, including smoking or excessive alcohol consumption (Verger et al., 2009). Both mechanisms acting together–improved health resulting from a more positive evaluation of high wellbeing or poorer health resulting from a more negative evaluation of low wellbeing–would be expected to result in a stronger association between wellbeing and health in more individualistic cultures.

In addition to differences in the appraisal of wellbeing, cross-cultural differences in the determinants of wellbeing could modify the association between wellbeing and health. Specifically, this association should be stronger in cultures where the determinants of wellbeing are more closely linked to good physical health. However, as the determinants of wellbeing in individualistic cultures (e.g. self-esteem) and collectivistic cultures (e.g. social ties) are both associated with favourable health outcomes and behaviours (Holt-Lunstad et al., 2010; Stamatakis et al., 2004), it is unclear which pattern of association should result in a stronger link between wellbeing and health.

Although previous studies have demonstrated that there are differences in the association between affect and health across cultures (Curhan et al., 2014; Miyamoto et al., 2013), these studies do not rule out possible confounds, including, for example, country-level differences in demographics, access to health care, life expectancy, diet, gross domestic product (GDP), and genetic make-up. This is highlighted by a recent study that found that cross-sectional associations between positive affect and self-rated health were stronger in low GDP countries (Haiti, Rwanda, Nigeria, Sierra Leone and Malawi) than in high GDP countries (United States, Ireland, Switzerland, Austria and Japan) (Pressman et al., 2013).

For our study, we tested whether cross-cultural differences in the degree to which countries were individualistic led to differences in the association between wellbeing and self-rated health (a subjective health measure) or mortality risk (an objective health measure). Specifically, considering the emphasis placed on wellbeing in individualistic cultures, we predicted that the association between wellbeing and health would be stronger in more individualistic countries. To rule out competing, non-cultural hypotheses to the greatest extent possible, we took two steps. First, we examined the strength of these associations across only European countries as doing so enabled us to compare countries that varied in their levels of individualism versus collectivism, but which were comparable in other factors. For instance, although Greece is a highly collectivistic country (even more so than Japan) and Italy is a individualistic country (only slightly less so than the United Kingdom) (Hofstede et al., 1991), Greece and Italy are similar in terms of their health care systems (Health Consumer Powerhouse, 2006), average life expectancies (Jakubowski et al., 1998) and diet (Trichopoulou et al., 2002). Second, we statistically controlled for differences in socio-economic status, education, health behaviours and country-level differences in healthcare provision.

Our study improved on previous research in another way, too. Previous cross-culture studies on wellbeing and health have been cross-sectional (Pressman et al., 2013). Consequently, it is unclear whether between country differences in the association reflect differences in how affect impacts physical health or vice versa. The present study, on the other hand, was based on longitudinal data. We therefore were able to test the association between wellbeing and subsequent mortality over a 10-year period, and control for baseline chronic disease prevalence.

## Methods

### Study population

30,816 participants aged 50 and over who lived in Austria, Belgium, Denmark, France, Germany, Greece, Israel, Italy, the Netherlands, Sweden, Switzerland, and Spain were recruited in the first wave of the Survey of Health Ageing and Retirement in Europe (SHARE) in 2004/2005. Since then, participants have been interviewed biennially. The SHARE project has been reviewed and approved by the Ethics Committee of the University of Mannheim (Alcser et al., 2005).

### Wellbeing

Wellbeing at wave 1 was assessed with the CASP-12, which is an abridged version of the CASP-19 (Hyde et al., 2003), which was developed to measure the wellbeing of the SHARE sample. The CASP-12 asks participants to indicate the frequency with which 12 statements (e.g., I look forward to each day) apply to their life. Responses on these statements were made on a four point Likert scale ranging from ‘never’ to ‘often’. The raw CASP-12 wellbeing scores therefore ranged from 0 to 48 with higher scores indicating higher wellbeing. For the 13,596 participants in our study sample, internal consistency for the CASP-12 was high (*α* = 0.83). CASP-12 scores were relatively stable over approximately 9 years: the re-test correlation coefficient between scores at wave 1 and wave 5 was *r* = 0.52, *p* < 0.001.

### Country individualism

We assigned each of the 11 European countries an individualism score according to Hofstede et al. (2010) cultural dimension of individualism (see Table 1).

**Table 1:** Country Individualism Scores

Country	Individualism score	Individualism tertile
Greece	35	Low
Spain	51	Low
Austria	55	Low
Germany	67	Low
Switzerland	68	Moderate
France	71	Moderate
Sweden	71	Moderate
Denmark	74	Moderate
Belgium	75	High
Italy	76	High
Netherlands	80	High

### Self-rated health

At wave 1, participants were asked to report whether their health was ‘very good’, ‘good’, ‘fair’, ‘bad’ or ‘very bad’. As only a small number of participants described their health as very bad (n = 178), we grouped these participants and participants who described their health as bad in the same category. Categories were coded 1 to 4 with 1 representing very good health and 4 representing bad or very bad health. For the analyses, self-rated health was treated as an ordered categorical variable.

### Mortality

Participant deaths were recorded from wave 1 onwards. Deaths were confirmed by a proxy respondent (family member, a household member or a neighbour) who reported the date and main cause of death. The categories for cause of death included cancer, heart attack, stroke, other cardiovascular related illness, respiratory disease, disease of the digestive system, severe infectious disease, accident and ‘other’.

### Confounding variables

We adjusted for several variables that might confound the association between wellbeing and mortality risk. These included age, sex, socioeconomic status (SES), level of education, depressive symptoms, marital status and history of cancer, heart attack, stroke, diabetes, chronic lung disease or any long-term health problems including long term illness, disability or infirmity. Older age, male gender, lower socioeconomic status and history of chronic disease or long term disability are established mortality risk factors (Case & Paxson, 2005; Kesteloot & Huang, 2003; Krieger et al., 1997; Majer et al., 2011; Riley & Cowan, 2014; Sorlie et al., 1995). Moreover, previous studies have documented an association between negative affect and mortality (Saz & Dewey, 2001) as well as an association between marital status and mortality (Johnson et al., 2000). Age, sex, socioeconomic status, level of education, depressive symptoms, marital status and history of chronic disease or long term health problems have also been related to subjective wellbeing (Brett et al., 2012; Hanmer et al., 2006; Pinquart & Sörensen, 2000; Pressman & Cohen, 2005; Steptoe et al., 2014; Wikman et al., 2011).

Socioeconomic status was indexed by total household assets, gross value of home, value of other real estate, value of any share of business and value of any vehicles minus mortgage of main residence. For the purposes of the analyses, we divided the sample into quintiles according to total household wealth. Using the International Standard Classification of Education (ISCED-97) framework, participants’ educational achievement was categorised according to their highest level of education: Pre-primary or primary, lower secondary, upper or post-secondary and first or second stage tertiary. To assess history of chronic illness, participants were asked whether a doctor had ever told them that they had any of the following conditions: ‘a heart attack including myocardial infarction or coronary thrombosis or any other heart problem including congestive heart failure’, ‘a stroke or cerebral vascular disease’, ‘diabetes or high blood sugar’, ‘chronic lung disease such as chronic bronchitis or emphysema’, ‘cancer or malignant tumour, including leukaemia or lymphoma, but excluding minor skin cancers’. Participants were additionally asked whether they had ‘any long-term health problems including illness, disability or infirmity’. The EURO-D was used to assess symptoms of depression (Prince et al., 1999). The scale consists of 12 items taken from the Geriatric Mental State scale (Copeland et al., 1986). Finally, participants were asked to report their marital status as ‘living with spouse’, ‘living with partner’ or ‘living as a single’. We used these responses to create two categories: living alone and living with partner or spouse.

To control for country-level differences in healthcare provision, we obtained each country’s health consumer index score from the Health Consumer Powerhouse (2006) report. This report assigns countries scores based on 28 indicators, including access to treatment, waiting times and health outcomes. Higher scores indicate a higher quality healthcare system; the health consumer index scores in our sample ranged from 576 (France) to 471 (Italy), the mean score was 517, which was closest to the score for Belgium (533).

### Mediating variables

We chose health behaviours (physical activity, alcohol consumption and smoking status) and body mass index (BMI) as potential mediators of the relationship between wellbeing and mortality risk. Both BMI and health behaviours have been associated with mortality risk (Ford et al., 2011; Prospective Studies Collaboration, 2009) and wellbeing (Pressman & Cohen, 2005; Rippe et al., 1998; Sjöström et al., 1992; Steptoe et al., 2014).

Participants reported the frequency with which they engaged in vigorous and or moderate physical activity using one of 4 responses: ‘more than once a week’, ‘once a week’, ‘one to three times a month’ and ‘hardly ever or never’. Responses were dichotomised based on activity frequency—either once a week (or more) or less than once a week. Responses were summed to create 3 categories: physical inactivity, moderate but not vigorous activity at least once a week and vigorous physical activity at least once a week. Participants reported their frequency of alcohol consumption as ‘5 days a week or more’, ‘1–4 days a week’, ‘twice a month or less’ or ‘not at all’. Participants reported their smoking status as non-smoker, former smoker or current smoker. BMI (kg/m^2^) was derived from participant self-reported height and weight, and participants were categorised as underweight (below 18.5), normal weight (18.5–24.9), overweight (25–29.9) or obese (30 or above).

### Statistical analysis

We first tested whether the cross-sectional association between wellbeing and self-rated health varied across countries that differed in individualism. To these ends we used an ordinal logistic regression with self-rated health as the outcome. We also included a term for the individualism score × wellbeing interaction in the model. A significant interaction would support the hypothesis that the association between wellbeing and self-rated health varies as a function of individualism. This model was additionally adjusted for age, sex, socioeconomic status and health care index.

Cox proportional hazards regression was used to examine the association between wellbeing at baseline and mortality over the follow-up period. Inspection of Schoenfeld residuals suggested that the proportional hazards assumption was not violated (all *p* values >0.1). We also tested for multicollinearity by calculating a variance inflation factor for each of the predictor variables in our model. This was achieved by regressing each predictor variable in turn on all remaining predictor variables and then using the R^2^ value to calculate the variance inflation factor using the formula 1/(1 − R^2^). All variance inflation factor scores were below 3 indicating that there was no multicollinearity. Survival time in days was calculated from the wave 1 interview date to the date of death or, for participants who did not die over the follow-up, the date of last follow-up interview.

We tested whether the association between wellbeing and mortality varied according to country individualism score by including the individualism score × wellbeing interaction in a model that adjusted for age, sex, socioeconomic status and health care index score.

We adjusted for confounding and mediating variables in three stages. In the first stage, we adjusted for age and sex in the model. In the second stage, we adjusted for age, sex, and the potential confounds (socioeconomic status, country level health care index score, level of education, depressive symptoms, marital status and history of cancer, heart attack, stroke, diabetes, chronic lung disease or any long-term health problems). In the third stage, we additionally adjusted for potentially mediating variables (smoking status, physical activity, alcohol consumption and BMI).

To test whether individualism moderated the association between wellbeing and cause-specific mortality, we repeated the cox proportional hazards regression replacing all-cause mortality with mortality from cardiovascular disease or cancer.

### Analytical sample

Of the 30,816 participants, 13,596 were included in the analysis. Participants were excluded if they had missing data for wellbeing (n = 12,140; 39%) or had missing data on any of the covariates (n = 5079; 27%). Participants excluded because they were missing wellbeing data were older, more likely to be female, were less physically active, consumed less alcohol, were less likely to smoke, had fewer years of education and were more likely to report a history of diabetes, stroke or heart attack. These participants also had a higher depression score and were lower in socioeconomic status. Analysis comparing baseline covariates between participants with available and unavailable vital status data at wave 5, indicated that, compared with participants with vital status data, participants with missing vital status had significantly lower wellbeing, lower depressive symptoms score, lower SES, fewer years of education, were more physically active, were more likely to be a current smoker, drink more alcohol and were less likely to report a history of cancer, heart attack or long-term illness.

11,595 participants were included in analysis predicting mortality from cardiovascular disease and 12,691 for analysis predicting mortality from cancer. Participants were excluded from these analyses if they reported a history of the relevant chronic disease at baseline: history of cardiovascular disease and cancer, respectively.

Finally, to test for possible bias due to missing data, we used multiple multivariate imputation to impute values of covariates with missing values using IBM SPSS Statistics 21 software. This approach assumes that data are missing at random, that is, the pattern of missingness is systematic and can be predicted by observed data (Garson, 2015). We assumed data were missing at random as missingness was significantly correlated with other measured variables (Garson, 2015). The imputation models included survival time, all-cause mortality and the covariate variables. Missing data were imputed for the sample of participants that took part at wave 1. We generated 35 imputed datasets using chained equations imputation.

## Results

Table 2 shows the baseline characteristics of the sample (n = 13,596) according to wellbeing tertile. Overall, people with higher wellbeing were younger, had lower depressive symptom scores, were wealthier, were more likely to be male, were less likely to be overweight, were more physically active, consumed more alcohol, were less likely to be a current smoker, were more educated, were less likely to live alone and were less likely to report a history of chronic disease or long-term illness (with the exception of history of cancer, which was not associated with wellbeing).

**Table 2:** Baseline characteristics stratified according to tertiles of wellbeing score (low, moderate and high subjective wellbeing) total n = 13,596

Characteristics	Lowest tertile	Middle tertile	Highest tertile	p-trend^a^
Age (years), M (SD)	65 (10)	63 (10)	62 (9)	<0.001
EURO-D score Mdn (IQR)	3 (1–5)	2 (1–3)	1 (0–2)	<0.001
Total wealth (€) M (SD)	327,000 (1,319,842)	624,000 (2,318,549)	844,100 (2,523,084)	<0.001
Female, No. (%)	2611 (57)	2725 (53)	2070 (53)	<0.001
BMI (kg/m^2^) M (SD)				<0.001
Underweight	72 (2)	61 (1)	27 (1)	
Normal weight	1586 (34)	1942 (38)	1743 (45)	
Overweight	1920 (42)	2277 (44)	1604 (41)	
Obese	978 (21)	859 (17)	528 (14)	
Physical activity, No. (%)				<0.001
Physically inactive	876 (19)	422 (08)	211 (05)	
Moderate physical activity	1767 (39)	1897 (37)	1246 (31)	
Vigorous physical activity	1913 (42)	2820 (55)	2445 (62)	
Alcohol consumption, No. (%)				<0.001
5 days a week or more	1087 (24)	1301 (25)	1106 (28)	
1–4 days a week	912 (20)	1583 (31)	1332 (34)	
Twice a month or less	933 (20)	1143 (22)	745 (19)	
Not at all	1624 (36)	1112 (22)	719 (18)	
Smoking status, No. (%)				<0.001
Non smoker	2465 (54)	2564 (50)	1931 (49)	
Former smoker	1156 (25)	1546 (30)	1228 (31)	
Smoker	935 (21)	1029 (20)	743 (19)	
Education, No. (%)				<0.001
Pre-primary or primary	2048 (45)	1367 (27)	716 (18)	
Lower secondary	838 (18)	939 (18)	740 (19)	
Upper or post-secondary	1155 (25)	1701 (33)	1342 (34)	
First or second stage tertiary	515 (11)	1132 (22)	1104 (28)	
Self-rated health, No. (%)				<0.001
Very good	395 (9)	1011 (20)	1361 (35)	
Good	1644 (36)	2603 (51)	1948 (50)	
Fair	1769 (39)	1292 (25)	533 (14)	
Bad or very bad	747 (16)	233 (5)	60 (2)	
Living alone, No. (%)	1331 (29)	1135 (22)	740 (19)	<0.001
History of heart attack, No. (%)	707 (16)	555 (11)	280 (07)	<0.001
History of stroke, No. (%)	227 (05)	148 (03)	74 (02)	<0.001
History of diabetes, No. (%)	521 (11)	394 (08)	263 (07)	<0.001
History of cancer, No. (%)	253 (06)	271 (05)	192 (05)	0.43
History of chronic lung disease, No. (%)	331 (07)	214 (04)	109 (03)	<0.001
Long term illness or disability, No. (%)	2656 (58)	2325 (45)	1438 (37)	<0.001

^a^Statistical significance is based χ^2^ tests or one-way ANOVA, as appropriate

Firstly, we tested whether the association between wellbeing and self-rated health was consistent across cultures. Of the 13,596 participants in our study sample, 2767 reported having very good health, 6195 reported having good health, 3594 reported having fair health and 1040 reporting having bad or very bad health. The wellbeing × individualism interaction was significant (*p* < 0.001). To illustrate this interaction, we divided the sample into tertiles according to the individualism of the country in which they lived (see Table 1 for a summary of countries in each tertile) and conducted an analysis for each group separately (see Table 3). In the sex- and age-adjusted models, the association between higher wellbeing score and better self-rated health was significant for all three groups; however, this association was weaker in the lowest individualism tertile (OR = 0.50; 95% CI = 0.47–0.52) compared with the moderate (OR = 0.38; 95% CI = 0.35–0.41) and high (OR = 0.41; 95% CI = 0.38–0.43) individualism tertiles. The association between wellbeing and self-rated health remained significant in the fully-adjusted model. Again, this association was weakest in the lowest individualism tertile (OR = 0.62; 95% CI = 0.60–0.66) compared to the moderate (OR = 0.55; 95% CI = 0.50–0.60) and high (OR = 0.57; 95% CI = 0.53–0.61) individualism tertiles.

**Table 3:** Proportional odds ratios (95% confidence intervals) of worse self-rated health according to a SD increase in wellbeing score, n = 13,596

Model	Individualism tertile	OR (95% CI)
Age and sex	Low	0.50 (0.47–0.52)**
	Moderate	0.38 (0.35–0.41)**
	High	0.41 (0.38–0.43)**
Confounding and mediating variables^a^	Low	0.62 (0.60–0.66)**
	Moderate	0.55 (0.50–0.60)**
	High	0.57 (0.53–0.61)**

*p* for wellbeing score × individualism score interaction <0.001

^a^Confounding variables = socioeconomic status, country level health care index score, level of education, depressive symptoms, marital status and history of chronic disease or any long term health problems. Mediating variables = health behaviours and BMI

* *p* < 0.05; ** *p* < 0.001

Next, we examined the association between wellbeing and mortality risk. 1405 deaths were reported between wave 1 and wave 5. The interaction between individualism score and wellbeing was not significant (*p* = 0.15); consequently, we report HRs for the whole sample. Table 4 displays hazard ratios (HRs) for all-cause mortality according to a standard deviation (SD) increase in wellbeing. In the age and sex adjusted model a SD increase in wellbeing was associated with a 22% decrease in mortality risk. This association remained significant although attenuated following adjustment for potentially confounding variables (depressive symptoms, socioeconomic status, health care index score, education, marital status and history of chronic disease or long-term illness), HR = 0.87; 95% CI = 0.82–0.93 and additional adjustment for potentially mediating variables (health behaviours and BMI), HR = 0.92; 95% CI = 0.86–0.97. In addition to wellbeing, younger age, being female, no history of chronic disease or long term illness, having a BMI of 18.5 kg/m^2^ or above (compared with a BMI below 18.5), being a non-smoker (compared with current smoker) and engaging in moderate or vigorous activity were all associated with reduced mortality risk.

**Table 4:** Hazard ratios (95% confidence intervals) for all-cause mortality according to a SD increase in wellbeing score, n = 13,596

Model	HR (95% CI)
Age and sex	0.78 (0.74–0.82)**
Confounding variables^a^	0.87 (0.82–0.93)**
Confounding and mediating variables^b^	0.92 (0.86–0.97)*

*p* for wellbeing score × individualism score interaction = 0.15

^a^Confounding variables = socioeconomic status, country level health care index score, level of education, depressive symptoms, marital status and history of chronic disease or any long term health problems

^b^Mediating variables = health behaviours and BMI

* *p* < 0.05; ** *p* < 0.001

There were 274 cardiovascular disease related deaths over the follow-up period. Our analysis revealed that the wellbeing × individualism interaction was significant (*p* = 0.01). Consequently, we divided the sample into tertiles according to individualism score and conducted analysis for each group separately. Table 5 displays HRs for mortality from cardiovascular disease according to a SD increase in wellbeing in each tertile. In the sex and age adjusted model, the association between wellbeing and cardiovascular mortality risk was significant for all three groups. A SD increase in wellbeing was associated with a 16% (HR = 0.84; 95% CI = 0.72–0.98) decrease in cardiovascular mortality risk in the tertile with low individualism scores, a 25% (HR = 0.75; 95% CI = 0.58–0.97) decrease in cardiovascular mortality risk in the tertile with moderate individualism scores and a 39% (HR = 0.61; 95% CI = 0.50–0.76) decrease in cardiovascular mortality risk in the tertile with high individualism scores. After adjustment for potentially confounding variables, this association remained significant only in the high individualism tertile; a SD increase in wellbeing was associated with a 37% (HR = 0.63; 95% CI = 0.49–0.81) decrease in cardiovascular mortality risk. Further adjustment for potentially mediating variables only attenuated this association slightly: HR = 0.64; 95% CI = 0.50–0.83. In addition to wellbeing, significant predictors of reduced cardiovascular mortality risk in the fully adjusted model in all three tertiles included: younger age and being female. Additional factors associated with a reduced risk in the low individualism tertile were no history of diabetes, living with a partner, engaging in vigorous or moderate physical activity and drinking twice a month or less (compared with drinking daily or almost daily). Additional factors in the moderate individualism tertile were not abstaining from alcohol (compared with drinking daily or almost daily) and being a non-smoker or former smoker. Finally, additional factors in the high individualism tertile were regular vigorous physical activity (compared with physical inactivity) and being a non-smoker (compared with being a current smoker).

**Table 5:** Hazard ratios (95% confidence intervals) for mortality from cardiovascular disease according to a SD increase in wellbeing score, n = 11,593

Model	Individualism tertile	Cases/N	HR (95% CI)
Age and sex	Low	133/4340	0.84 (0.72–0.98)*
	Moderate	67/3431	0.75 (0.58–0.97)*
	High	74/3822	0.61 (0.50–0.76)**
Confounding variables^a^	Low		0.90 (0.75–1.08)
	Moderate		0.74 (0.55–1.01)
	High		0.63 (0.49–0.81)**
Confounding and mediating variables^b^	Low		0.95 (0.79–1.15)
	Moderate		0.81 (0.60–1.10)
	High		0.64 (0.50–0.84)**

*p* for wellbeing score × individualism score interaction = 0.007

^a^Confounding variables = socioeconomic status, country level health care index score, level of education, depressive symptoms, marital status and history of chronic disease or any long term health problems

^b^Mediating variables = health behaviours and BMI

* *p* < 0.05; ** *p* < 0.001

358 cancer related deaths were reported over the follow-up period. The association between wellbeing score and cancer mortality risk was not significant in the age and sex adjusted model (HR = 0.91; 95% CI = 0.82–1.00), the model adjusted for confounding variables (HR = 0.95; 95% CI = 0.84–1.07) or the model adjusted for confounding and mediating variables (HR = 0.99; 95% CI = 0.88–1.12). The wellbeing × individualism interaction was also not significant (*p* = 0.60).

To test for possible bias due to missing data, we used multiple multivariate imputation to impute values of covariates with missing values. The pooled effect sizes from analysis with imputed information were similar to those obtained from analysis predicting risk of all-cause mortality employing the sample with complete data. These results therefore suggest that missing covariate data did not bias the results. See supplementary Table 1 for a comparison of results.

## Discussion

We tested whether associations between wellbeing and self-rated health or mortality risk were consistent among people from individualistic and collectivistic cultures. Our results were mixed. In cross-sectional analysis, higher wellbeing was more strongly related to better self-rated health among people from individualistic cultures. In predicting all-cause mortality, however, we found that higher wellbeing was associated with a reduced risk, but individualism did not moderate this effect. Analyses of cause-specific mortality, on the other hand, revealed a significant association between higher wellbeing and reduced risk of mortality from cardiovascular disease, which was significantly stronger among participants in countries scoring high on individualism. Wellbeing was not associated with risk of cancer related mortality.

The stronger link between wellbeing and self-rated health or cardiovascular mortality in more individualistic countries, suggests that these associations differ between cultures. By comparing only European countries and controlling for differences in health care provision, SES, education and health behaviours, we were able to rule out the effect of multiple between country differences not directly related to the cultural dimension of individualism. Our findings are in line with previous cross-sectional studies that found a stronger link between negative affect and number of chronic conditions or levels of interleukin-6 in American (individualistic) compared with Japanese (collectivistic) samples.

Various mechanisms might explain why there is a stronger link between wellbeing and health in individualistic compared with collectivistic cultures. Firstly, as outlined in the Introduction, this effect may reflect the greater emphasis placed on wellbeing in individualistic cultures. High wellbeing may lead to more positive emotion in individualistic cultures as it is valued. This more positive evaluation may confer an additional health benefit. Furthermore, an emphasis on personal wellbeing in individualistic cultures, may cause individuals with low wellbeing to feel distressed, which in turn, may impact negatively on health.

Although numerous authors have reported that wellbeing is valued more highly in individualistic than collectivistic cultures (Ahuvia, 2002; Diener & Suh, 2000; Steptoe et al., 2007; Veenhoven, 1999), others have argued that these findings reflect a failure to measure wellbeing in collectivistic cultures (Uchida et al., 2004). Wellbeing is commonly defined as a cognitive and affective appraisal of the quality of one’s own life (Diener, 2000). Uchida et al. (2004) argue that this definition is valued equally across cultures; however, there are likely to be cultural differences regarding which factors an individual considers when appraising their quality of life. Norasakkunkit and Kalick (2002) point out that the majority of wellbeing measures currently used in psychological research assess factors that are prioritised in individualistic but not collectivistic cultures (e.g. autonomy, personal success). The CASP-12 (wellbeing measure) is a good example of this bias: participants rate the extent to which they feel autonomous and have control over their lives. Although these are important correlates of wellbeing from an individualist perspective, they are unlikely to constitute ‘a good life’ in collectivistic cultures. Bearing this criticism in mind, the current results (stronger association between CASP-12 score and self-rated health or mortality risk from cardiovascular disease in individualist countries) could reflect a failure to capture wellbeing among participants from more collectivistic cultures.

Even if our measure of wellbeing was not culturally biased, cross-cultural differences in the determinants of wellbeing could account for the moderating effect of culture on the association between wellbeing and risk of cardiovascular disease mortality. Specifically, the stronger association in more individualistic countries could reflect the fact that wellbeing is more dependent on good physical health or health related variables in these cultures. In this sense, ratings of wellbeing could function as an index of physical health in these cultures and thus be more closely related to subsequent health outcomes. In support of this argument, in our study, wellbeing was more strongly related to self-rated health in more individualistic countries.

Although wellbeing was more strongly related to self-rated health and cardiovascular related mortality in individualistic cultures, the association between wellbeing and risk of all-cause mortality did not vary as a function of individualism. It is unclear why we found evidence of an interaction between wellbeing and level of individualism in analysis predicting mortality from cardiovascular disease but not from all causes. It may be that the mechanisms that underlie the association between wellbeing and causes of death other than cardiovascular disease are less dependent on cultural context. However, further work is needed to confirm this effect.

Wellbeing score was not associated with cancer mortality risk. Previous findings regarding the association between wellbeing and cancer risk have been mixed. Some studies have documented a significant association between wellbeing and cancer in women (Feller et al., 2013; Wakai et al., 2007), but we failed to find any association between wellbeing and incident cancer in the ELSA sample (n = 7460) (Okely & Gale, 2016). Similarly, Lillberg et al. (2002) found no association between wellbeing and risk of breast cancer in a Finnish cohort.

Strengths of the study include the sample—which was large and representative of people aged 50 and older living in Europe. The available data allowed for adjustment for many potential confounders and mediator variables. However, several limitations should be noted. Firstly, over a third of the participants (39%) were excluded due to missing wellbeing data. Excluded participants differed to those included in our sample on a number of covariate variables. Thus, excluding these participants may have biased the results; however, analysis with imputed missing covariate and wellbeing data yielded similar effect sizes to those obtained for the sample with complete data, suggesting that this exclusion did not bias our results. Secondly, date and cause of death was obtained from interviews with a relative or friend of the participant rather than from official death records and may therefore be less reliable. Thirdly, a significant proportion of participants had missing mortality data. At wave 2, information on vital status could not be obtained for 19% of participants in our sample, by wave 5, 38% had missing vital status data. The effect of bias due to these missing data cannot be ruled out. Finally, although we controlled for between-country differences in socio-economic status, education, some health behaviours and healthcare provision, it is possible that additional unmeasured differences between more and less individualistic countries (e.g. in diet or health literacy) may account for the apparent effect of individualism in our study.

We hope that our findings will inspire further investigation into the association between wellbeing and health across cultures. Researchers could test whether the previously documented association between wellbeing and incident cardiovascular disease (Boehm & Kubzansky, 2012) is also stronger in individualistic than in collectivistic cultures. Wellbeing scales oriented towards more collectivist values are now being developed (Datu et al., 2016). It would be interesting to test whether our finding of a stronger link between wellbeing and self-rated health or risk of mortality from cardiovascular disease in more individualist cultures would be replicated if the CASP-12 was replaced with a more collectivist measure of wellbeing. It is possible that the opposite effect would be found-with stronger associations between ‘collectivist wellbeing’ and health in more collectivist cultures.

To conclude, although previous studies have documented cross-cultural difference in the association between negative affect and health (Curhan et al., 2014; Miyamoto et al., 2013), our findings regarding the link between wellbeing and health were mixed. We found no evidence of a moderating effect of individualism score in analysis predicting all-cause mortality. However, our results did provide some evidence that individualism moderates the association between wellbeing and self-rated health or risk of mortality from cardiovascular disease. Although prospective studies are needed to confirm our finding, our work illustrates the importance of incorporating cultural context into the study of wellbeing and health.

## Electronic supplementary material

Below is the link to the electronic supplementary material.

Supplementary material 1 (DOCX 14 kb)
