# Supplementary material for: The interaction between individualism and wellbeing in predicting mortality: Survey of Health Ageing and Retirement in Europe
Source: J Behav Med. 2017 Jul 15;41(1):1–11. doi: 10.1007/s10865-017-9871-x (PMC5765189; doi:10.1007/s10865-017-9871-x)
Supplement: Supplementary file 1 — Supplementary material 1 (DOCX 14 kb) [file 10865_2017_9871_MOESM1_ESM.docx]

| **Supplementary Table 1.** *Hazard ratios (95% confidence intervals) from analysis with imputed missing covariates and from analysis with complete data* | | |
| --- | --- | --- |
| Model | Imputed Covariates  HR (95%-CI) | Complete Data  HR (95%-CI) |
| age and sex | 0.76 (0.72-0.78)** | 0.78 (0.74-0.82)** |
| confounding and mediating variables ^a^ | 0.92 (0.87-0.97)* | 0.92 (0.87-0.98)* |
| ^a^ Confounding variables = socioeconomic status, country level health care index score, level of education, depressive symptoms, marital status and history of chronic disease or any long term health problems. Mediating variables = health behaviours and BMI  * *p* <0.05, ** *p* <0.001 | | |
